# Plasmonic Refractive Index Sensor with High Figure of Merit Based on Concentric-Rings Resonator

**DOI:** 10.3390/s18010116

**Published:** 2018-01-04

**Authors:** Zhaojian Zhang, Junbo Yang, Xin He, Jingjing Zhang, Jie Huang, Dingbo Chen, Yunxin Han

**Affiliations:** 1 College of Liberal Arts and Sciences, National University of Defense Technology, Changsha 410073, China; 376824388@sjtu.edu.cn (Z.Z.); 15574944061@163.com (J.Z.); huangjie@nudt.edu.cn (J.H.); c_dingbo@163.com (D.C.); 2 Center of Material Science, National University of Defense Technology, Changsha 410073, China; xinhestudy@163.com (X.H.); hanyx15@163.com (Y.H.)

**Keywords:** plasmonic sensor, high figure of merit, concentric double rings resonator

## Abstract

A plasmonic refractive index (RI) sensor based on metal-insulator-metal (MIM) waveguide coupled with concentric double rings resonator (CDRR) is proposed and investigated numerically. Utilizing the novel supermodes of the CDRR, the FWHM of the resonant wavelength can be modulated, and a sensitivity of 1060 nm/RIU with high figure of merit (FOM) 203.8 is realized in the near-infrared region. The unordinary modes, as well as the influence of structure parameters on the sensing performance, are also discussed. Such plasmonic sensor with simple framework and high optical resolution could be applied to on-chip sensing systems and integrated optical circuits. Besides, the special cases of bio-sensing and triple rings are also discussed.

## 1. Introduction

Surface plasmon polaritons (SPPs) are electromagnetic fields propagating along the interface of metal-insulator, and have been widely discussed for several decades due to the ability to modulate light in nanoscale, as well as break the diffraction limit [1]. Recently, many kinds of plasmonic devices have been investigated, such as filters [2], absorbers [3], splitters [4], and sensors [5]. Among these, plasmonic sensors have drawn more attention, because when compared with traditional optical sensors, such as fiber sensors and silicon-based sensors, plasmonic sensors have much smaller size with comparable sensing performance, which means that they are more suitable for integrating [6]. Various of plasmonic sensors have come out lately such as refractive index sensors [7], temperature sensors [8], phase sensors [9], and gas sensors [10].

The metal-insulator-metal (MIM) waveguide is one of the basic plasmonic waveguides with the capability to confine light within considerable propagating length [11], and many works of sensor are based on this structure [12,13,14,15,16,17,18,19,20]. As a sensor, it requires both high sensitivity (S) and high figure of merit (FOM) to promise an excellent performance with high optical resolution. Great efforts have been made to improve sensitivity on MIM plasmonic sensors, but longer wavelength always suffers from wider FWHM [12,13,14,15], which means lower FOM. Recently, Fano resonance has been universally applied to enhance FOM that results from structure symmetry break or dark-bright resonance interference, and it has an asymmetric spectral line shape with narrower FWHM [16,17,18,19,20]. But such resonance is unstable and can be easily broken due to phase or mode mismatch, which could be caused by localized corrosion of structure, change of localized refractive index, and so on.

In this study, a traditional MIM plasmonic sensor, coupled with simple concentric double rings resonator (CDRR), is proposed. Utilizing the special supermodes, the FOM of that can be significantly improved to as high as 272.3 in the visible-light region without Fano resonance, as well as 203.8 in the near-infrared region. Such value of FOM is quite larger than other recent works [5,7,12,13,14,15,16,17,18]. The peculiar properties of CDRR supermodes and the impact of structure parameters on sensing performance are also discussed. The two-dimensional (2D) Finite-Difference Time-Domain (FDTD) solution is used to simulate this structure with perfectly matched layer (PML) boundary condition.

## 2. Structural Model and Theory Analysis

**Figure 1:**
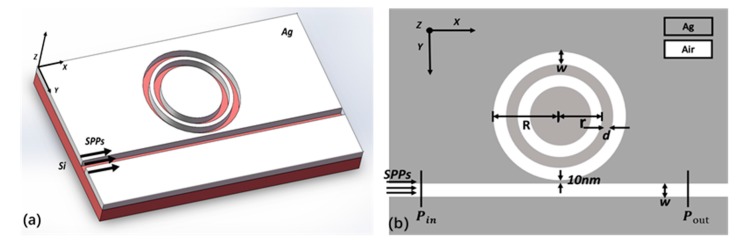
(**a**) The three-dimensional (3D) picture of the plasmonic sensor. (**b**) The two-dimensional (2D) picture of the plasmonic sensor.

As shown in Figure 1, a MIM waveguide coupled with a CDRR is proposed. The silicon substrate is plated with silver, which is chosen for the relative low loss [21]. The waveguides are etched on the silver surface. Such a structure can be fabricated by the method of focused ion beam (FIB) [22]. As shown in 2D picture, the grey and white areas represent silver and air, respectively. The permittivity of air is set as $\varepsilon_{i}=1$, as for silver (Ag) the Drude model is utilized as follows [2]:

(1) $$\varepsilon_{m}=\varepsilon_{\infty }-\frac{\omega_{p}^{2}}{\omega (\omega +i\gamma )}$$

Here $\varepsilon_{\infty }$ gives the medium constant for the infinite frequency, $\omega_{p}$ refers to bulk frequency for plasma, γ means damping frequency for electron oscillation, and ω shows incident light angular frequency. The parameters for silver are $\varepsilon_{\infty }$ = 1, $\omega_{p}$ = 1.37 × $10^{16}$ Hz, and γ = 3.21 × $10^{13}$ Hz. 

In this structure, R = 350 nm and r = 270 nm is the radius of the outer and inner ring, respectively. The width of the waveguide is set as w = 50 nm, and the gap between inner and outer ring is d = 30 nm. The gap between CDRR and bus waveguide is g = 10 nm. To collect the incident and transmitted power, two monitors are put, respectively, at $P_{in}$ and $P_{out}$. The transmittance of power can be calculated by $T\;=\;P_{out}/P_{in}.$

TM mode SPPs can propagate at the surface of interface in the waveguide when coupled into the MIM structure. When compared with incident wavelength, the width of the bus waveguide is much smaller, so fundamental TM mode can exist only. The dispersion relation of this fundamental mode is described as follows [11]:

(2) $$\frac{\varepsilon_{i}p}{\varepsilon_{m}k}=\frac{1-e^{kw}}{1+e^{kw}}$$

(3) $$k=k_{0}\sqrt{{\left(\frac{\beta_{spp}}{k_{0}}\right)}^{2}-\varepsilon_{i}},p=k_{0}\sqrt{{\left(\frac{\beta_{spp}}{k_{0}}\right)}^{2}-\varepsilon_{m}}$$

(4) $$\beta_{spp}=n_{eff}k_{0}=n_{eff}\frac{2\pi }{\lambda }$$

Here, w refer to the width of bus waveguide, λ shows incident light wavelength in vacuum, $\varepsilon_{i}$ and $\varepsilon_{m}$ give the relative dielectric and metal permittivity, $\beta_{spp}$ and $n_{eff}$ are propagation constant and effective refractive index of SPPs, and $k_{0}=2\pi /\lambda$ means wave number.

**Figure 2:**
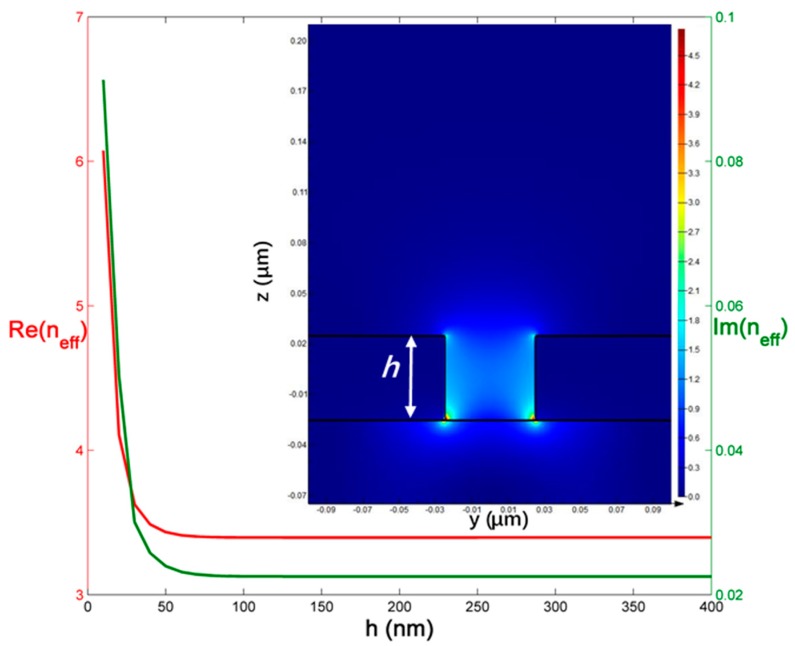
The relationship between the effective refractive index of the surface plasmon polaritons (SPPs) and the height of the waveguide at wavelength 850 nm. The inset is the distribution $\left|P_{x}\right|$ of the fundamental mode at 850 nm when *h* = 50 nm.

Most of the proposed and designed MIM plasmonic devices have used 2D simulation, which is infinite in one dimension, to test the performance of the devices with much shorter simulation time and lower loss [23], and so is this work. But, the height of the waveguide has a significant effect on the loss of the system, which has to be considered for practical processing. Here, the relationship between the effective refractive index of the SPPs and the height of the waveguide at 850 nm is shown in Figure 2. As the height increases, both the real and imaginary part of the $n_{eff}$ will decrease at start, which is due to the decrease of the fraction corresponding to the modal power in the metal at interfaces. The reduction of the imaginary part of $n_{eff}$ means less loss, which can lead to longer propagation length [24]. However, when greater than a certain height, $n_{eff}$ will remain stable. If continuing improving the height, $n_{eff}$ will approach the value in 2D simulation, which is the h→∞ case. The distribution $\left|P_{x}\right|$ of the fundamental mode at 850 nm when *h* = 50 nm is presented in the inset of Figure 2. At other wavelengths, the outcome will be similar. After comparing the values at different wavelengths, the most suitable height can be selected. Our result is agreed with [24]. 

**Figure 3:**
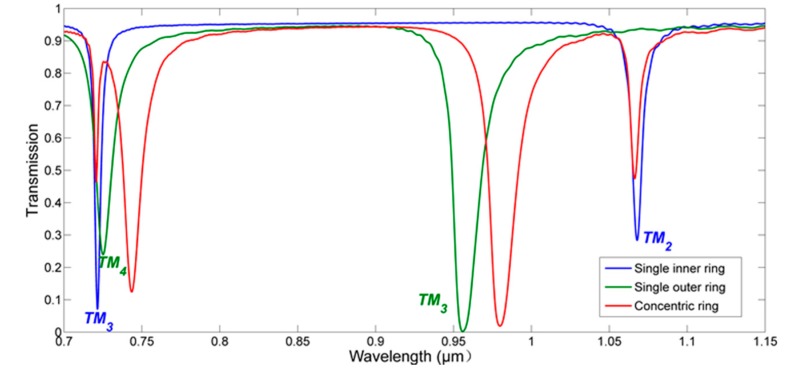
The transmission spectra of metal-insulator-metal (MIM) waveguide coupled with single inner (outer) ring or concentric double rings resonator (CDRR).

The transmission spectra of MIM waveguide coupled with single inner (outer) ring or CDRR are shown in Figure 3. In order to illuminate the principle of CDRR better, the gap between bus waveguide and single inner (outer) ring is set as 30 nm (10 nm). Figure 3 indicates that the transmission spectrum of CDRR is almost the superposition of the other two spectra with slight shifts, and the FWHM of the mode near 1060 nm becomes narrower. For a single ring resonator, the resonance condition can be described as [5]:

(5) Nλ=Re(neff)Leff,N=1,2,3...

where $n_{eff}$ refers to the effective refractive index of the ring resonator, which can be solved by Equations (2)–(4), $L_{eff}$ means effective perimeter, generally refers to the average of the inner and outer perimeters. N refers to mode number which is an integer.

For the CDRR, the resonance condition is the superposition of that for single ring, and the shift of resonant wavelength comes from the change of effective index of each ring resulting from the interaction between each other. 

**Figure 4:**
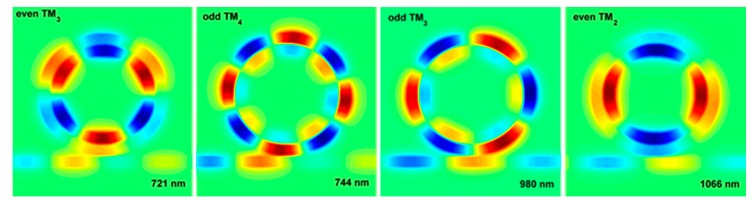
The supermodes of CDRR.

It is found that the modes of CDRR exist in the form of supermodes, which means that the inner and outer ring have resonance simultaneously. The novel supermodes of CDRR are introduced, as shown in Figure 4. However, we notice that there are only two kinds of supermodes: when the energy is mainly trapped in outer ring, the mode in the inner ring will have anti phase; when mainly trapped in inner ring, it is in phase. Refer to the supermodes between two parallel waveguides [25]; the two supermodes could be called odd mode (with anti-phase) and even mode (with in-phase). The mechanism of such supermodes can be explained by the coupled-mode theory (CMT) [26]. The propagation constant in the ring resonator can be derived with Equation (5):

(6) Re(β)=2πRe(neff)λ=2πλ⋅Nλ2πReff=NReff

where $R_{eff}$ is the effective radius of the ring resonator, and the propagation constant of odd (even) mode in the system of two parallel single-mode waveguides is as follows [26]:

(7) $$\beta_{odd}=\bar{\beta }-\Delta \beta$$

(8) $$\beta_{even}=\bar{\beta }+\Delta \beta$$

(9) $$\bar{\beta }=\frac{\beta_{1}+\beta_{2}}{2},\Delta \beta =\frac{1}{2}\sqrt{4K^{2}+{\left(\beta_{1}-\beta_{2}\right)}^{2}}$$

Here, $\bar{\beta }$ represents the average propagation constant, $\beta_{1}$ ($\beta_{2}$) is the propagation constant for a single waveguide without coupling, and *K* is the coupling coefficient. In the condition of the codirectional coupling, *K* is a pure imaginary number [26]. Equations (7) and (8) shows that the Re(βeven) is always larger than Re(βodd). Since the CDRR could be considered as two parallel bend waveguides with periodicity, this mode expression can also be applied. Now, the CDRR system is considered as a kind of single-ring resonator, the effective radius of which is determined by the energy distribution. When the energy is mainly trapped in the outer side, the effective radius is larger, leading to smaller propagation constant according to Equation (6), bringing about the odd mode with anti-phase in the CDRR. Otherwise, it will be an even mode with in-phase.

**Figure 5:**
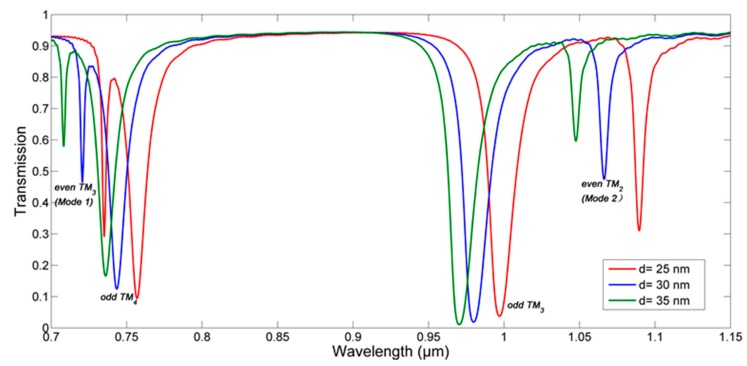
The transmission spectra correspond to different gaps.

**Figure 6:**
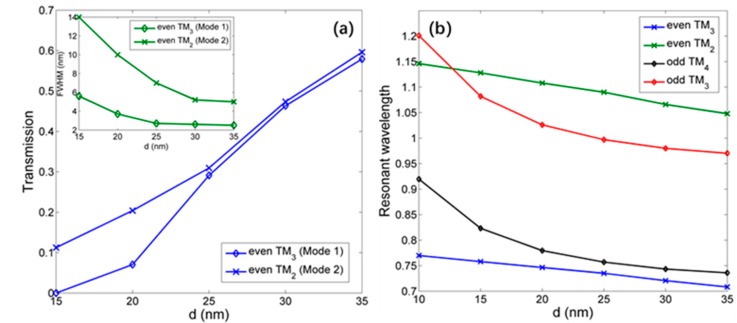
(**a**) The relationship between Transmission (FWHM) and gap. (**b**) The relationship between resonant wavelength and gap.

By changing the radius of inner ring (which also means the change of the gap *d*), the transmission spectra correspond to different gaps is obtained in Figure 5. Obviously, the resonant wavelength will have a blue shift when increasing the gap. The Figure 6b shows the details, and we find that for even modes, the resonant wavelength has a linear relationship with gap; for odd modes, it is nonlinear. It can be explained by the effective index. When changing the radius of inner ring linearly, the effective index of outer ring will be influenced and have a nonlinear change, leading a nonlinear relationship between odd-mode (which can be regarded as mode of inner ring approximately according to the energy distribution) resonant wavelength and gap. However, the inner ring will suffer both radius and gap change, and that will keep its effective index unchanged, bringing about the linear relationship between even-mode resonant wavelength and the radius of inner ring as Equation (5). Such conclusion can be useful for designing the CDRR.

Apparently, the even mode has much narrower FWHM; therefore, the two even modes (even $TM_{3}$ and $TM_{2}$) are investigated next, which is assumed as mode 1 and mode 2, respectively. Figure 6a shows the relationship between gap d and FWHM (transmission). When increasing the gap, the FWHM of even mode will be narrower, meanwhile suffering from the increase of transmission. It is due to the special mode distribution of even mode, the energy of which is mainly trapped in the inner ring, and the outer ring suppresses the energy loss, which is beneficial for energy storage. As the inner and outer rings have the same symmetry, the supermode is stable and has the single-ring mode features. Such even modes are potential to generate ultra-high Q factors, which is defined as Q=λ/FWHM. With gap 30 nm, the Q for mode 1 can reach 277 and for mode 2 is 205. The Q will be larger with increasing gap, however there is a trade-off between Q and transmission.

## 3. Refractive Index Sensing

**Figure 7:**
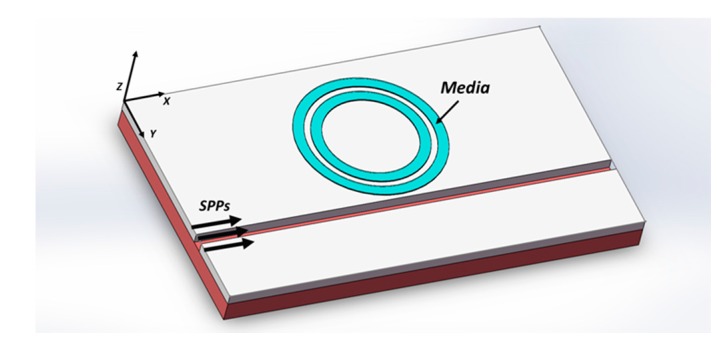
The 3D picture of the plasmonic sensor filled will media.

**Figure 8:**
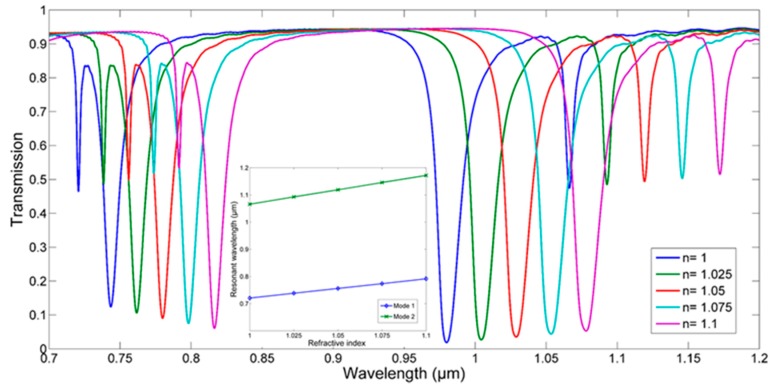
The transmission spectra with different filling dielectric in the CDRR. The inset shows the relationship between refractive index and resonant wavelength.

When considering the balance between FWHM and transmission, the gap d is chosen to be 30 nm. The plasmonic sensor filled with media is shown in Figure 7, and Figure 8 presents the transmission spectra with different filling dielectric in the CDRR. The refractive index of dielectric is increased from 1 to 1.1 with the step 0.025, leading to a red shift of the spectra. The sensing capabilities is defined as follows [27]:

(10) $$S=\frac{△\lambda }{△n},\mathrm{FOM}=\frac{S}{\mathrm{FWHM}}$$

The sensitivity S is described as the resonant wavelength shift when the dielectric has a unit change. Since the optical resolution is also crucial for sensors, a high figure of merit (FOM) is needed. By fitting the line in the inset of Figure 8, the sensitivity 708 nm/RIU of mode1 and 1060 nm/RIU of mode 2 are obtained. The FWHM of each is 2.6 nm and 5.2 nm, leading to the high FOM, which is 272.3 and 203.8, respectively. It must be mentioned that such value of FOM is quite larger than other recent works, as shown in Table 1. Although some works achieved a higher figure of up to hundreds of thousands [19,20], the FOM definition of these works is different, which always refers to ${\mathrm{FOM}}^{*}$ [27].

**Figure 9:**
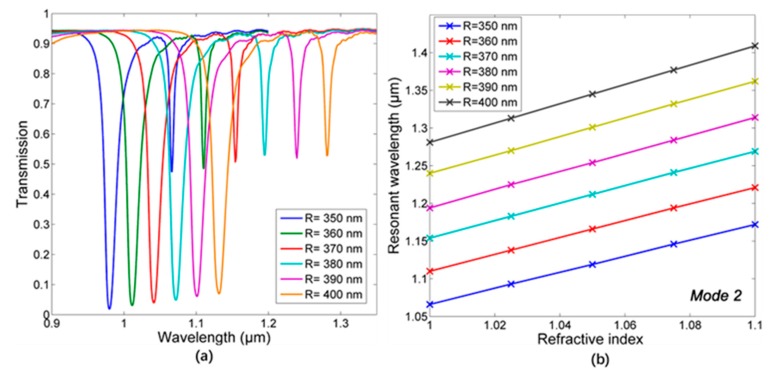
(**a**) The spectra with different radius of CDRR. (**b**) The ling fitting between refractive index and resonant wavelength.

Due to the higher sensitivity with excellent FOM in the near-infrared region, mode 2 is further studied then. The radius of CDRR is increased with the gap d fixed at 30 nm, and the spectra are given in Figure 9a. When the outer radius raises from 350 nm to 400 nm with the interval 10 nm, the sensitivity is 1112 nm/RIU, 1152 nm/RIU, 1196 nm/RIU, 1224 nm/RIU, and 1280 nm/RIU, respectively, from Figure 9b, and FOM is 185.3, 164, 149.5, 153, and 150.4, respectively. Obviously, increasing the size of CDRR can improve the sensitivity performance of the sensor with the cost of lower FOM, which may, respectively, arise from the longer optical path and higher dissipation of energy.

**Figure 10:**
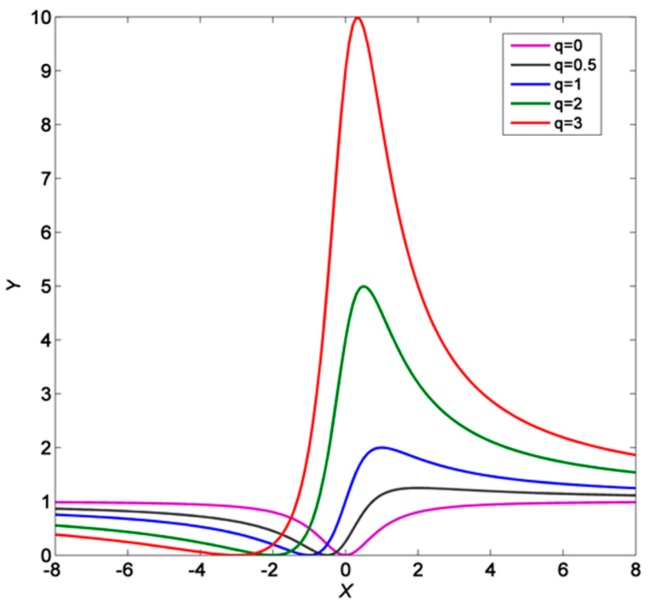
The Fano spectrum shape corresponding to different *q* values.

Comparing with Fano resonance, which is the hotspot for sensing recently [27], this structure has superiority in stability. Although Fano resonance has a narrow FWHM leading to high FOM, few works pay attention to the instability of this state. Fano resonance occurs when a discrete state interferes with a continuum band of states, the shape of the special transmission spectrum can be described by Fano formula [28]:

(11) $$Y=\frac{{\left(q+X\right)}^{2}}{1+X^{2}}$$

where q=cotδ is the Fano parameter, δ is the phase shift of the continuum between two modes, depending on geometric and material parameters of the system [28], which can be caused by corrosion, fabrication errors, change of localized refractive index, and so on. Figure 10 presents the spectrum shape corresponding to different *q* values. Due to the character of cot function, little phase shift will cause significant change of *q*, leading to the breaking of the high-*q* Fano shape, and then bring about the decline of sensing performance. 

**Figure 11:**
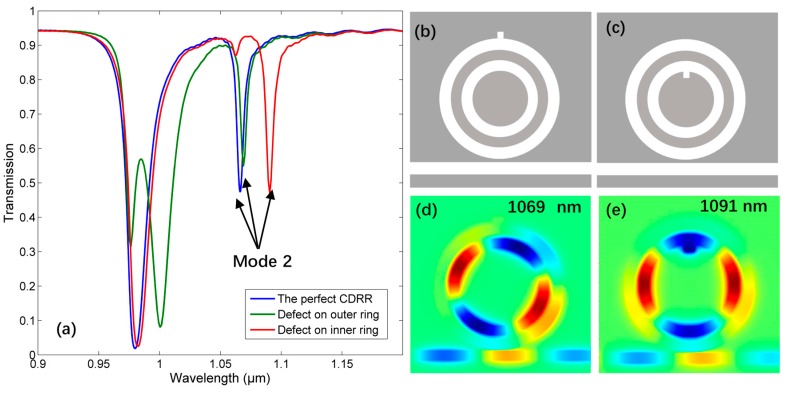
(**a**) The transmission spectrum of mode 2 with defect. (**b**) CDRR with defect on outer ring. (**c**) CDRR with defect on inner ring. (**d**) The $\left|H_{z}\right|$ of mode 2 with defect on outer ring. (**e**) The $\left|H_{z}\right|$ of mode 2 with defect on inner ring.

The supermode in CDRR is based on traditional micro-ring resonance, which is not that fragile. To test the stability of this sensor, a 5 nm×5 nm square defect is inserted at two different positions of CDRR, respectively, as given in Figure 11b,c. From the corresponding transmission spectrum of mode 2 shown in Figure 11a, the defect on outer (inner) ring only has influence on the resonance of outer (inner) ring, which can make resonant wavelength shift or create new mode. However, the mode 2 still exists with narrow FWHM in both cases, only suffers from little lower absorption or resonant wavelength change. The corresponding distributions $\left|H_{z}\right|$ of mode 2 with defect are presented in Figure 11d,e.

## 4. Bio-Sensing

**Figure 12:**
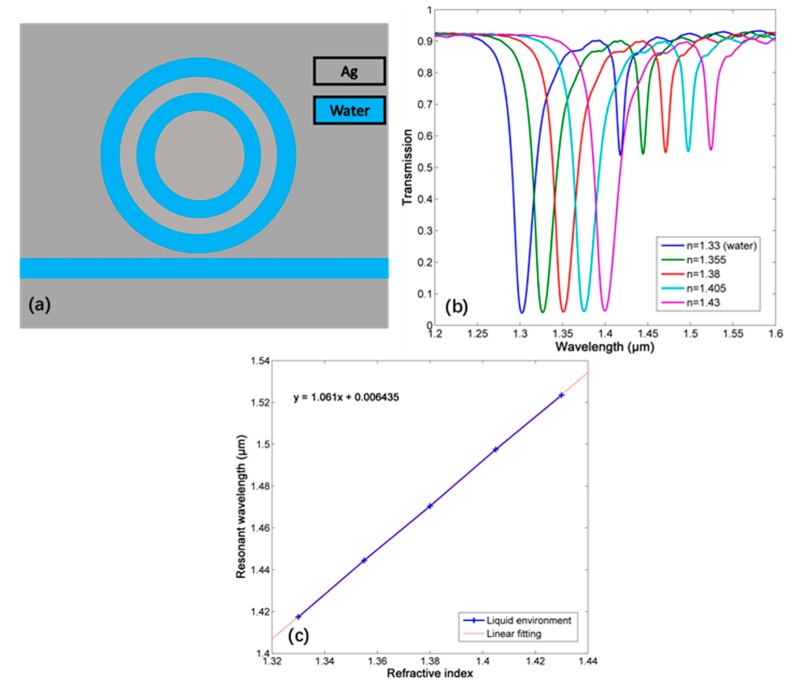
(**a**) Sensor at water. (**b**) The transmission spectrum of sensor in liquid environment. (**c**) The relationship between refractive index and resonant wavelength of mode 2.

Here, the application for bio-sensing is discussed, the same structure parameters are chosen with outer ring radius 350 nm and gap 30 nm, and still only mode 2 is focused. Since water is one of the most common solvents used in chemical and biological applications, it is necessary to test the performance of the sensor in water (*n* = 1.33), as presented in Figure 12a. When considering the concentration of solution can be reflected by the refractive index (RI), a set of different RI values around the water are used to measure the sensitivity and FOM, the transmission spectrum is given in Figure 12b. According to Figure 12c, the sensitivity in the water is 1061 nm/RIU, and the FOM of 193 can be achieved, which can prove that this sensor still has excellent performance in water.

**Figure 13:**
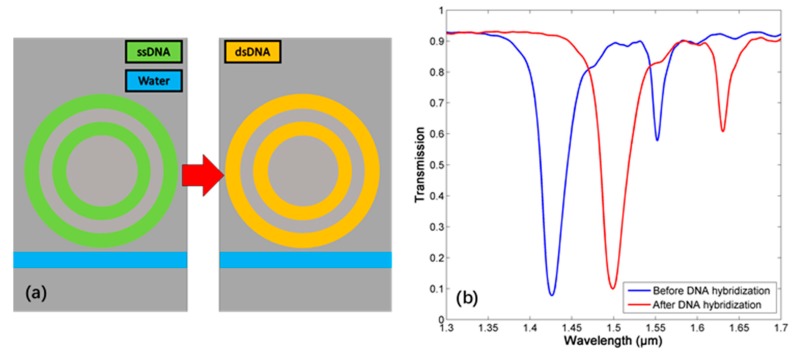
(**a**) The process of DNA hybridization. (**b**) The transmission spectrum before and after DNA hybridization.

Therefore, such structure can be a sensitive label-free and compact biosensor. A special case for the detection of the DNA hybridization is shown as follows. After the DNA hybridization, i.e., when single strand DNA (ssDNA) becomes double strands DNA (dsDNA), the RI of DNA layers will change from 1.456 to 1.53 [29]. If a layer of ssDNA is implanted inside the resonator, the DNA hybridization can be detected, as shown in Figure 13. After the hybridization, the resonant wavelength of mode 2 shifts from 1553 nm to 1631 nm with high optical resolution, which can be probed easily.

**Figure 14:**
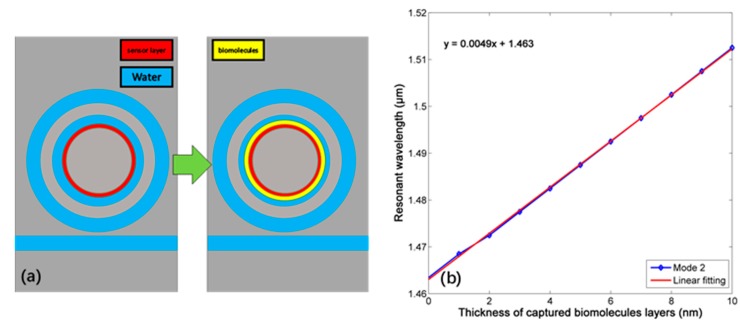
(**a**) The selective biochemical process. (**b**) The relationship between resonant wavelength and thickness of captured layer.

Another bio-application of this sensor is to detect the biomolecules attached to the inner wall of the resonator. We assume that a combined sensor layer of thickness $t_{cap}$, which consists of an activating intermediate layer and a capture layer, has been immobilized onto the wall of inner ring, which can capture a layer of biomolecules of thickness $t_{bio}$ through a selective biochemical process, such as antigen-antibody binding process [30,31,32], as shown in Figure 14a. The RI and material dispersion of the biolayers will depend on how the biomolecules are oriented. Here, we neglect the dispersion of the biolayers and assume that they have the same RI of 1.45, which is close to that of silica [31], which is a realistic assumption that is proved by experimental measurements with MOF biosensors [33]. Assuming $t_{cap}=10\;\mathrm{nm}$, the relationship between resonant wavelength and thickness of captured layer $t_{bio}$ is shown in Figure 14b, and a thickness sensitivity ($S^{\prime }=\Delta \lambda /\Delta t)$ of 4.9 nm/nm can be gained. When comparing with traditional micro-structured optical fiber (MOF) biosensors [33], this plasmonic biosensor has comparable sensitivity, higher optical resolution and much smaller footprint. It has to be mentioned that from Figure 12b and Figure 13b, the FWHM of mode 2 can remain narrow within a wide environment effective RI range. Since layers effect can be equal to the change of effect RI essentially [34], the detection for the layer can still possess the character of high optical resolution.

**Figure 15:**
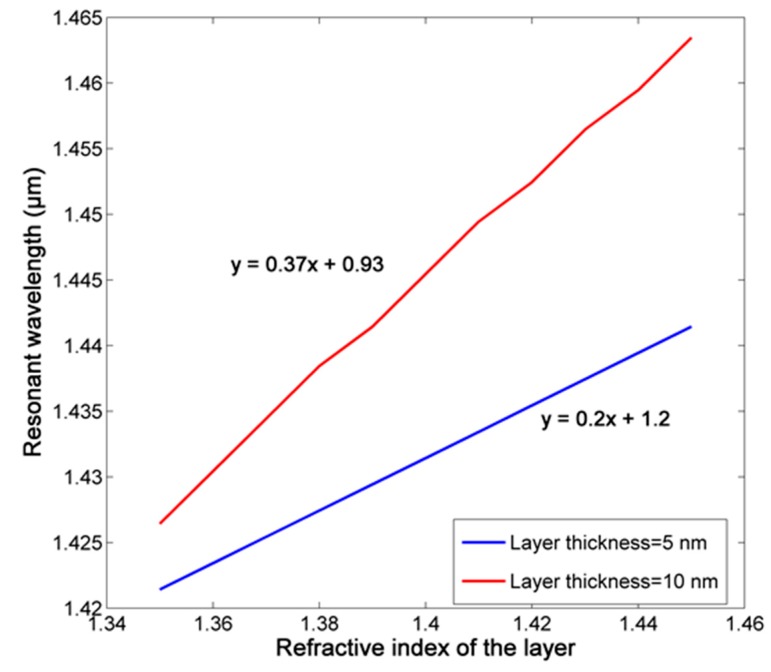
The relationship between layer refractive index (RI) with certain thickness and resonant wavelength.

Finally, when considering a biomolecules layer attached to the wall of inner ring with a certain thickness *t*, the sensitivity for the layer RI is measured for the case *t* = 5 nm and *t* = 10 nm, shown as Figure 15. We choose both sizes because these are the most common feature scale of single biomolecule [32], and the sensitivity 200 nm/RIU (*t* = 5 nm) and 370 nm/RIU (*t* = 10 nm) can be realized with high optical resolution.

## 5. Case of Triple Ring

**Figure 16:**
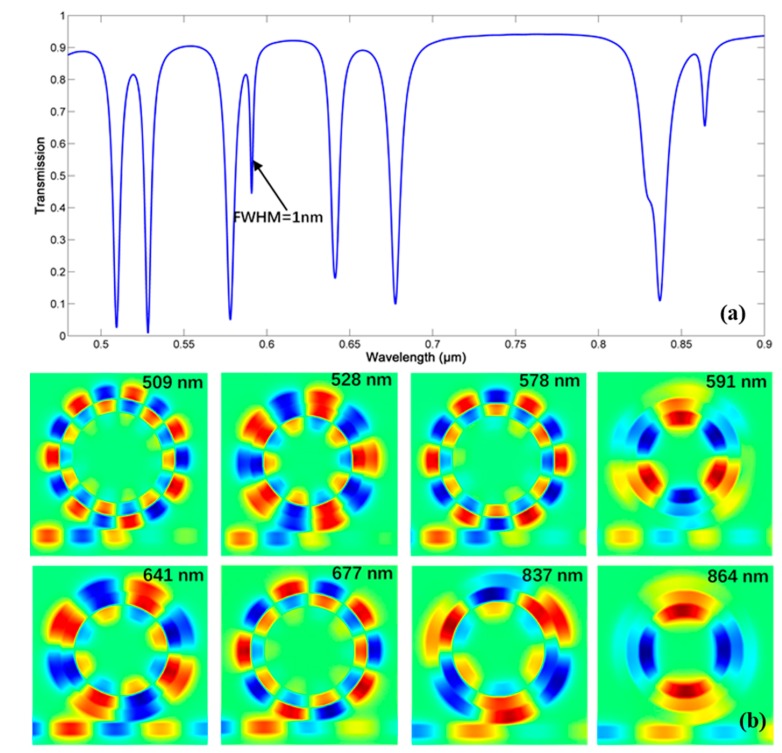
(**a**) The transmission spectrum. (**b**) The mode distribution of concentric triple rings resonator (CTRR).

Out of interest, the case of concentric triple rings resonator (CTRR) is proposed with *w* = 50 nm, *d* = 15 nm, *g* = 10 nm, and *R* = 350 nm. The transmission spectrum and mode distribution are presented in Figure 16. Here, assuming that the binary *0*/*1* represent a set of opposite phases, and that the array *[1 b c]* describes the supermode (we set outer-ring-phase as 1), where *b*, *c* give the phase of mode in middle ring and inner ring. After checking the supermodes for a wide wavelength range, we found that only three kinds supermodes exist: *[1 1 0]*, *[1 1 1]*, and *[1 0 1]*.

For supermode *[1 1 1]*, an ultra-narrow FWHM can be reached, as shown in Figure 16, when λ=591 nm and 864 nm. When λ=591 nm, the FWHM is nearly 1 nm, and an ultra-high Q = 591 can be gained. The reason is that in this supermode, most energy is trapped in the inner ring. Therefore, the outer double rings can suppress the energy loss. However, this supermode may suffer from the low energy absorption, such as that at 864 nm. Additionally, CTRR will possess a great many resonant wavelengths, it may bring about some disturbances on sensing. This structure may have potential applications to be found. 

## 6. Conclusions

In summary, a sensor based on MIM waveguide coupled with a CDRR is investigated in the near-infrared region, and a high sensitivity with ultra-high FOM is obtained in RI-sensing and bio-sensing. The special features of supermodes in CDRR is discussed, presenting that the even mode can produce ultra-narrow FWHM. The special case of triple rings is also discussed. In addition, such a structure also provides the concept to design a high Q microcavity. Utilizing the centrosymmetric characters of outer and inner microcavity, an ultra-high Q factor can be achieved without breaking the original mode characteristics. This device can also be used as multi band-stop filter in plasmonic system, which may have applications in integrated optical circuits. 

## Figures and Tables

**Table 1:** Comparison of sensitivity (S) and figure of merit (FOM) reported in various Plasmonic sensor.

Reference	S (nm/RIU)	FOM
Chen, L., et al. [5]	1496	124.6
Yan, Shu Bin, et al. [13]	868.4	43.9
Tang, Y., et al. [16]	1125	74
Zhang, Zhidong, et al. [17]	596	7.5
Zafar, Rukhsar, and Salim, M. [18]	1060	176.7
This paper	1060	203.8
